# Dizziness in the emergency department and risk of stroke: A systematic review and meta-analysis

**DOI:** 10.1371/journal.pone.0346556

**Published:** 2026-04-08

**Authors:** Haiyan Lin, Meier Zhu, Xiaoli Zhang, Yanliang Tang

**Affiliations:** 1 Department of Preventive Treatment, Hangzhou Fuyang Hospital of Traditional Chinese Medicine, Hangzhou, Zhejiang, China; 2 Department of Neurology, Hangzhou Fuyang Hospital of Traditional Chinese Medicine, Hangzhou, Zhejiang, China; Fondazione Policlinico Universitario Agostino Gemelli IRCCS, ITALY

## Abstract

**Background:**

Dizziness is a frequent emergency department (ED) presentation, and a subset of patients, especially those with isolated dizziness without focal neurological deficits, have stroke but are prone to misdiagnosis and adverse outcomes. Reported stroke prevalence in ED dizziness cohorts is highly heterogeneous, and comprehensive assessments of isolated dizziness remain limited.

**Methods:**

Following PRISMA guidelines, we systematically searched PubMed, Web of Science, Embase, and the Cochrane Library for relevant studies. We included cross-sectional studies reporting stroke prevalence among all ED patients with dizziness or isolated dizziness. A random-effects model was used for meta-analysis to calculate pooled prevalence. Subgroup analyses and Egger’s test were employed to explore heterogeneity and publication bias. The diagnostic accuracy of bedside diagnostic tools was also systematically reviewed.

**Results:**

Twenty-nine studies involving 161,013 ED patients presenting with dizziness were included. The pooled stroke prevalence among all ED dizziness patients (n = 158,583) was 5.5% (95% CI: 4.1–7.1). Among patients with isolated dizziness (n = 2,559), the pooled prevalence was 13.9% (95% CI: 8.2–20.9), substantially higher than in the overall dizziness cohort. Subgroup analyses indicated diagnostic methods and hospital level as major contributors to heterogeneity. Summary analysis of bedside diagnostic tools showed that HINTS and STANDING examinations have high diagnostic accuracy overall, while the TriAGe+ score can be applied flexibly for screening or confirmation based on different cut-off points.

**Conclusions:**

ED patients with dizziness carry a meaningful, setting-dependent stroke risk. Standardized bedside exams (HINTS, STANDING) improve early triage, and selectively deploying neuroimaging helps prevent missed strokes when risk is high.

## Introduction

Dizziness is among the most frequent reasons for emergency department (ED) presentation, accounting for roughly 3–4% of all visits [1–3]. The core challenge for clinicians is to identify patients with life-threatening central etiologies, particularly acute ischemic or hemorrhagic stroke [4,5]. A substantial proportion of posterior circulation (vertebrobasilar system) strokes present with dizziness, vertigo, or imbalance as the primary or even sole symptom [6–8]. Given that stroke remains a leading cause of disability and mortality worldwide [9], and that its management is highly time-sensitive, encapsulated by the concept “time is brain”, rapid and accurate ED triage is essential.

Missed or misdiagnosed strokes remain significant problems in ED practice [10,11]. The risk of error is markedly increased in patients presenting with only isolated dizziness – defined as dizziness without accompanying focal neurological deficits such as limb weakness, facial asymmetry, or slurred speech. These patients are easily misclassified as having benign peripheral vestibular disorders (e.g., benign paroxysmal positional vertigo or vestibular neuritis) [6,12]. Such misdiagnoses can delay revascularization therapies like thrombolysis or thrombectomy, with consequences that include catastrophic neurological injury or death and substantial healthcare costs [13,14]. Therefore, precisely quantifying the risk of stroke among ED patients with dizziness, particularly those with isolated dizziness, is crucial for developing effective clinical screening strategies, optimizing healthcare resource allocation, and improving the quality and safety of ED care.

Numerous studies have investigated stroke prevalence among ED patients presenting with dizziness, yet the existing evidence shows marked heterogeneity and inconsistency. Reported prevalence rates vary widely, from less than 1% to over 10% [15–17]. This variation likely stems from multiple factors, including study design, population definitions, the diagnostic reference standard for stroke confirmation, and the level of the participating healthcare institutions. Furthermore, many studies also have limited sample sizes and do not systematically assess these potential sources of variability. Although prior systematic reviews exist, several are outdated or do not comprehensively incorporated recent evidence [18–20]. Rigorous quantitative analysis focused on the high-risk isolated dizziness subgroup remains insufficient.

To address these knowledge gaps, we conducted a systematic review and meta-analysis with the following objectives: (1) to comprehensively update and pool estimates of the prevalence of acute stroke among adult ED patients presenting with dizziness globally; (2) to specifically focus on the isolated dizziness subgroup and more precisely calculate their stroke prevalence; (3) to investigate potential sources of heterogeneity through detailed subgroup analysis and meta-regression; and (4) to systematically evaluate the diagnostic performance of commonly used bedside tools. This study aims to provide a comprehensive overview of the available epidemiological evidence on stroke risk among ED patients presenting with dizziness.

## Materials and methods

### Study protocol

This systematic review and meta-analysis adhered to the Preferred Reporting Items for Systematic Reviews and Meta-Analysis (PRISMA) guidelines (see PRISMA 2020 Checklist). The pre designed protocol for this study was registered on the Open Science Framework (OSF): https://doi.org/10.17605/OSF.IO/JY3UV.

### Literature search

We systematically searched PubMed (from inception to July 30, 2025), Web of Science, Embase, and the Cochrane Library (from inception to July 31, 2025). Our goal was to identify studies reporting stroke prevalence among all ED patients with dizziness or the subgroup with isolated dizziness. A combination of Medical Subject Headings (MeSH) and keywords was used to search the databases. The detailed search strategies are provided in S1 Table. To ensure inclusion of the most recent evidence, we performed an additional PubMed search on September 15, 2025, which identified one additional eligible study that was incorporated into the meta-analysis. We also performed a meticulous manual search of the reference lists of identified studies and relevant reviews to ensure that no pertinent articles were missed.

### Inclusion and exclusion criteria

Literature was screened based on the following criteria. Inclusion criteria: Population: All ED patients with dizziness, or patients with isolated dizziness; participants aged ≥16 years. Study populations were consecutively and without selection bias. Outcome: The study reported quantitative outcomes (number of dizzy patients, number or incidence of stroke). Language: No restrictions. Article Type: Cross-sectional studies. Publication Year: No restrictions. Region/Country: No restrictions.

Exclusion criteria: (1) Reviews, case reports, conference abstracts, editorials, and letters to the editor. (2) Duplicate publications or studies focusing on the same cohort of subjects. (3) Studies limited to specific populations, e.g., only elderly patients, only those undergoing MRI, or studies that excluded patients with peripheral vertigo. (4) Studies lacking clear information on their methods or outcomes relevant to the interest of this review.

Definitions: Dizziness patients were defined as those presenting to the ED with dizziness, vertigo, or unsteadiness as the primary complaint. We use dizziness as an umbrella term throughout the manuscript. Isolated dizziness patients were defined as those experiencing dizziness, vertigo, or imbalance in the absence of impaired consciousness and focal neurological deficits, including visual field defects, facial asymmetry, sensory loss, dysarthria, dysphagia, aphasia, diplopia, or unilateral limb weakness.

### Study selection and data extraction

Literature was managed using EndNote software. Two researchers independently screened studies according to the inclusion and exclusion criteria. Discrepancies were resolved by discussion and consensus. Non-English articles were translated using Google Translate if necessary. If studies involved overlapping patient populations, the study with the most detailed information was selected. A pre designed Excel form was used for data extraction. Two researchers independently extracted required data from the articles into the form. Data included: first author, publication year, study location (country), study period, data source (hospital type/level), sample size, number of stroke cases, participant age, gender, diagnostic method, imaging coverage rate, diagnostic efficiency of tools, etc. When the original article did not directly provide the number of male patients, we calculated it based on the total sample size and the number (or proportion) of female patients. For imaging coverage rate, we derived them from the total sample size and the number of examinations performed. Studies were categorized by the primary method used to confirm stroke outcomes: (1) MRI with or without follow-up (all patients underwent MRI); (2) clinical follow-up/composite assessment, which encompasses any combination of medical record review, post-discharge follow-up, linkage to registries, and adjudication of outcomes without mandatory MRI in all patients; and (3) coding/discharge summary only, in which stroke diagnosis was based solely on administrative data or ED discharge codes without further verification.

### Quality assessment

Study quality was independently assessed by two researchers using the Joanna Briggs Institute (JBI) Critical Appraisal tool for prevalence studies. A third researcher arbitrated disagreements. The JBI tool comprises nine criteria: (1) Was the sample frame appropriate to address the target population? (2) Were study participants sampled in an appropriate way? (3) Was the sample size adequate? (4) Were the study subjects and the setting described in detail? (5) Was the data analysis conducted with sufficient coverage of the identified sample? (6) Was the condition measured in a standard, reliable way for all participants? (7) Were valid methods used for the identification of the condition? (8) Was there appropriate statistical analysis? (9) Was the response rate adequate, and if not, was the low response rate managed appropriately? Each criterion was assigned a score of 1 for “Yes” and 0 for “No”. Based on the total score, each article was rated as high quality (≥8 points), moderate quality (6–7 points), or low quality (≤5 points).

### Statistical analysis

Continuous variables were presented as mean with standard deviations, while categorical variables were presented as counts and percentages. Pooled analysis of extracted data was performed using Stata software. Heterogeneity between studies was assessed using Cochran’s Q statistic and the I² statistic. Due to anticipated heterogeneity, a random-effects model was used for meta-analysis. Publication bias was examined using funnel plots and Egger’s test, with a *P* value less than 0.05 indicating statistical significance. Furthermore, subgroup analyses were conducted based on various factors, including publication year (≤ 2015 vs > 2015), geographic region (Europe, Asia, Oceania, North America), sample size (≥ 500 vs < 500; ≥ 300 vs < 300), study design (prospective vs retrospective), diagnostic method (MRI with/without follow-up; clinical follow-up/composite assessment; coding/discharge summary only), imaging coverage rate (100% vs < 100%/unspecified), and patient source (tertiary/teaching hospitals; stroke centers; community hospitals). A *P* value < 0.05 for between-subgroup differences was considered statistically significant.

## Results

### Study selection

**Fig 1:**
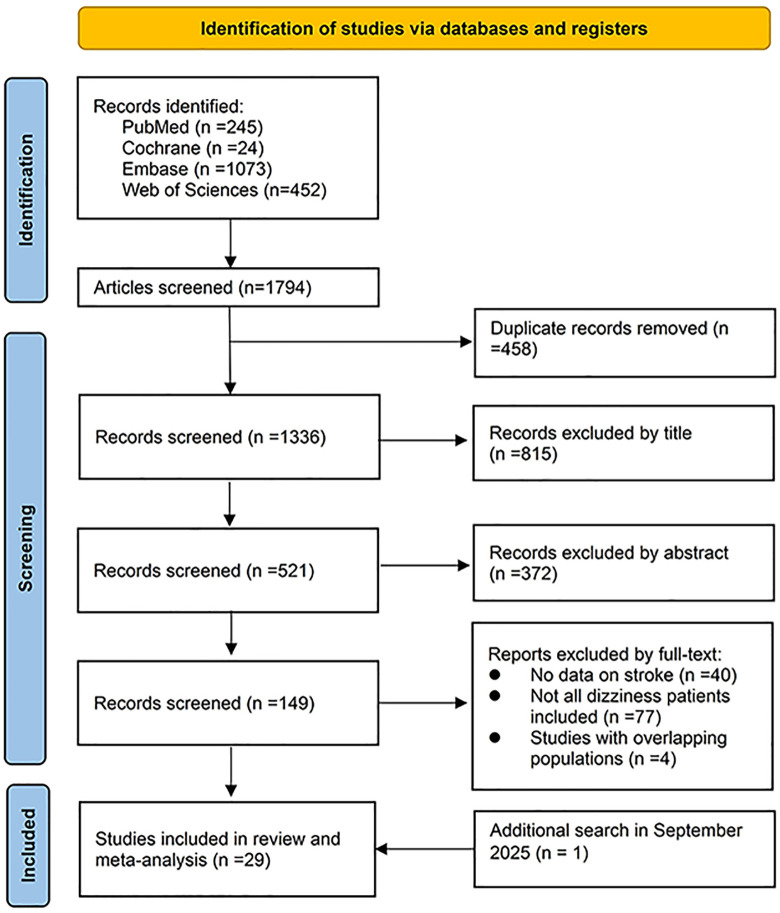
PRISMA flow of study selection.

Out of 1794 citations identified by the search strategy, 149 articles were considered relevant based on title and abstract screening. After full-text assessment, 29 manuscripts met the inclusion criteria [21–49]. The study selection process and reasons for exclusion are shown in Fig 1.

### Study characteristics

Baseline characteristics of the 29 included studies, encompassing 161,013 ED patients with dizziness, are summarized in Table 1. Publication dates ranged from 2006 to 2025. Among the included studies, 20 reported stroke prevalence in all ED dizziness patients, 9 reported stroke prevalence in isolated dizziness patients, and 1 study reported both cohorts. Geographically, most reports were conducted in Asia (12 studies) and North America (9 studies), followed by Europe (7 studies) and Oceania (1 study). Regarding patient source, most studies involved patients from tertiary/teaching hospitals (22 studies), followed by community hospitals (4 studies) and stroke centers (2 studies). Regarding diagnostic methods, most studies relied on clinical follow-up/composite assessment (19 studies), followed by MRI with or without follow-up (6 studies) and coding/discharge summary only (4 studies). Overall, 16 studies were of high quality, and 13 were of moderate quality (S2 Table).

**Table 1:** Baseline characteristics of the studies included in the systematic review.

Study	Year	CTRY	Design	Case	Dizz	Age(y)	Men(%)	Stroke, TIA n(%)	Source	Dx mthd	Image rate
Xiao Hu, 2025	Apr-Dec 2024	CHN	Retro	251	All Iso	63.0 (55.0-71.0)	120 (47.8)	/	Tea	C/C	92.0%
Robert Ohle, 2025	Jul. 2019- Aug. 2022	CAN	Pros	2078	All	77.1	1185 (39.8)	106 (5.1)	Tea	C/C	30.9%
Adrian Ho-Kun Yu, 2024	19 Jul. 2021−30 Sept. 2021	CHN	Retro	455	All	62	168 (36.9)	55 (12.1)	Tea	C/C	63.0%
Yu-Sung Chang, 2024	2012-2021	CHN	Retro	24266	All	59.0 ± 17.8	9277 (39.2)	1454 (6.1)	Tea	C/D	/
Lukas Comolli, 2023	Jul. 2015-Aug. 2020	CHE	Retro	1535	All	55.7 ± 18.6	745 (48.5)	/	Ter	C/C	69.9%
S Kmetonyova, 2023	Jan. 2020-Jun. 2021	CZE	Pros	140	All	56.8	45 (37.8)	/	Tea	MRI	100%
Arfa Samreen R, 2021	Mar. 2019-Mar. 2020	IND	Pros	70	All	58.69 ± 16.53	36 (51.4)	5 (7.1)	Ter	MRI	100%
Jonathan Hanna, 2019	Jan. 2014-Jun. 2018	USA	Retro	17884	All	55.1	6796 (38.0)	/	Tea	C/C	28.6%
Mürsel Koçer, 2019	Jan. 2013-Jan. 2015	TUR	Retro	5056	All	53.6 ± 18.2	2152 (42.6)	162 (3.2)	TR	C/D	59.2%
Micaela Ljunggren, 2018	1 Jan. 2012–31 Dec. 2014	SWE	Retro	2126	All	64 ± 19	916 (43.1)	330 (15.5)	Tea	C/C	44.8%
Yongwoo Kim, 2018	1 Jan. 2010−31 Dec. 2014	USA	Retro	77993	All	68.7 ± 15.9	33993 (43.58)	2394 (3.1)	Uns/ Mix	C/D	41.0%
Hussam Ammar, 2017	1 Jan 2011–31 Dec 2011	USA	Retro	521	All	49.3 ± 15.1	220 (42.2)	24 (4.6)	Tea	C/C	42.0%
Timothy McDowell, 2016	1 Jan 2011−31 Dec 2011	CAN	Retro	642	All	63	255 (39.7)	/	Ter	C/C	31.0%
Karen Chen, 2016	Jan 2012-Dec 2013	USA	Retro	1216	All	56 ± 19	508 (41.8)	/	Ter	C/C	/
Rui Felgueiras, 2014	1 Oct 1998− 30 Sept 2000	PRT	Pros	363	All	60.1 ± 16.4	134 (37.2)	/	Comm	C/C	63.3%
Babak B Navi, 2012	1 Jan 2007−31 Dec 2009	USA	Retro	907	All	59 ± 19	378 (41.7)	10 (1.1)	Ter	C/C	35.4%
Ching–Chih Lee, 2012	1 Jan 2004−31 Dec 2004	CHN	Retro	1118	All	/	395 (35.3)	/	Tea	C/D	/
Anthony S Kim, 2011	1 Jan 2008−31 Dec 2008	USA	Retro	20795	All	57 (42-72)	8280 (39.8)	/	Comm	C/C	27.2%
Yosuke Tona, 2011	1 Apr 2008–31 Mar 2009	JPN	Retro	650	All	57.0	256(39.38)	/	Ter	C/C	26.9%
C S K Cheung, 2010	24 May-24 Jun 2004	CHN	Pros	413	All	57	145(35.1)	26(6.3)	Tea	C/C	13.6%
Lam, J. M. Y., 2006	12-19 Dec 2003	CHN	Pros	104	All	/	34 (32.7)	1(1.0)	Tea	C/C	/
Maria Bijl, 2025	1 Jan 2016–1 Jan 2019, 13 Oct 2017−13 Oct 2020	NLD	Retro	500	Iso	/	221(44.2)	/	Tea	C/C	68.40%
Ayse Cagla Ozmert Toplu, 2025	2018- 2021	TUR	Pros	357	Iso	65.6 ± 14.4	30(51.7)	20(5.6)	SC	MRI	100%
Mattia Ronchetti, 2025	Jun 2022-Jun 2023	ITA	Pros	456	Iso	60(49-72)	171(37.5)	27(5.9)	Mix	C/C	62.9%
James Orton Thomas, 2022	Feb 2018- Sept 2021	AUS	Pros	133	Iso	62.0 ± 14.6	72(54.14)	14 (10.53)	SC	MRI	100%
Ebru Unal Akoglu, 2018	1 Nov 2010−30 Apr 2011	TUR	Pros	137	Iso	54.9 ± 15.6	56 (38.9)	/	Ter	MRI	95.10%
Simone Vanni, 2015	May 2011-Jan 2012	ITA	Pros	292	Iso	60 ± 16.3	42 (42.9)	/	Tea	C/C	57.90%
Aslı Gülfer Kartal, 2014	1 Jan 2011−30 Dec 2011	TUR	Pros	82	Iso	51	34 (41.5)	0	Ter	MRI	100%
Maureen Chase, 2014	1 Nov 2009−30 Oct 2010	USA	Pros	473	Iso	56.7 ± 19.3	181 (38.3)	37 (7.8)	Tea	C/C	/

TIA: transient Ischemic Attacks; MRI: magnetic resonance imaging; USA: United States of America;/: not available. Pros: Prospective; Retro: Retrospective; Iso: isolated; CTRY: Country; Turkey: TUR; China: CHN; Australia: AUS; Italy: ITA; Netherlands: NLD; Japan: JPN; Portugal: PRT; Sweden: SWE; Czech Republic: CZE; Switzerland: CHE; India: IND; Canada: CAN. Tea: Teaching hospitals. Ter: Tertiary hospitals. SC: Stroke center. Mix: Mixed hospital. Comm: community Hospitals. TR: Training and research hospital. Uns: Unspecified. C/C: Clinical follow-up/composite assessment. C/D: Coding/discharge summary only. MRI: MRI with or without follow-up. Dx: Diagnostic. Mthd: method. Dizz: Dizziness.

### Stroke prevalence in all ED dizziness patients

**Fig 2:**
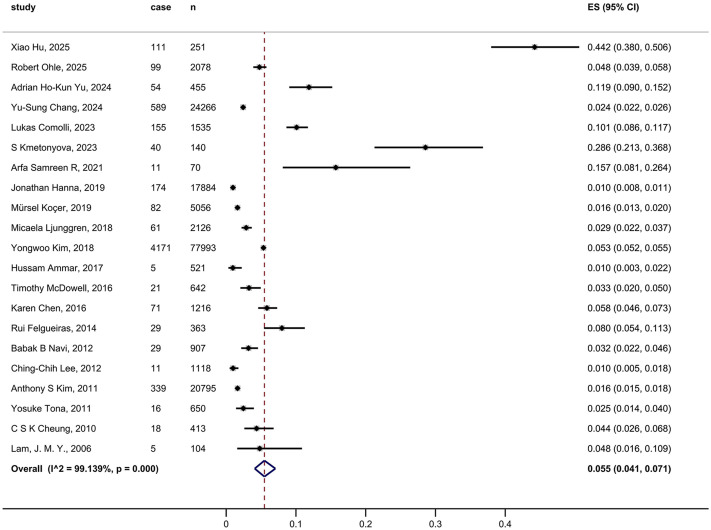
Pooled prevalence of stroke among all ED dizziness presentations.

Analysis of 21 studies involving 158,583 individuals showed that the overall pooled prevalence of stroke among all ED dizziness patients was 5.5% (95% CI, 4.1–7.1; I² = 99.14) (Fig 2). The funnel plot appeared asymmetric (S1A Fig). Egger’s test indicated significant publication bias among the 21 included studies (P = 0.088). Sensitivity analysis using the leave-one-out method did not reveal any single study whose exclusion caused a directional change in the results (S1B Fig).

Among these 21 studies, 12 reported the number of ischemic and hemorrhagic strokes separately. The pooled prevalence of ischemic stroke in all ED dizziness patients was 6.9% (95% CI, 4.9–9.1; I² = 98.91) (S2A Fig), while hemorrhagic stroke prevalence was 0.3% (95% CI, 0.1–0.4; I² = 78.88) (S2B Fig). Additionally, 12 studies reported the number of TIAs, yielding a pooled TIA prevalence of 2.2% (95% CI, 1.3–3.4; I² = 96.83) (S2C Fig).

### Subgroup analysis of stroke in all dizziness patients

The estimated stroke prevalence in all ED dizziness patients according to different subgroups was summarized (Table 2). Based on subgroup analysis, diagnostic method and imaging coverage rate were the most important sources of heterogeneity (P < 0.001). Studies using MRI (with or without follow-up) as the diagnostic method had the highest stroke prevalence about 24.0%. In contrast, studies relying on clinical follow-up/composite assessment and those using only coding/discharge summary had prevalence rates of only 5.5% and 2.4%, respectively. Similarly, studies with 100% imaging coverage had a significantly higher prevalence (24.0%) than those with inadequate or unspecified coverage (4.6%). Studies with smaller sample sizes (<500 participants) reported a significantly higher stroke prevalence (14.9%) than larger sample studies (≥500; 3.0%), with a between group difference *P* = 0.002. Studies published after 2015 reported higher stroke prevalence (6.9%) than those published in 2015 or earlier (3.1%; P = 0.003). Differences were observed between studies from different continents (P = 0.016). European studies had the highest prevalence (10.5%), followed by Asia (6.8%), with North America having the lowest (3.0%). Prospective designs showed higher prevalence (9.4%) than retrospective studies (4.5%; P = 0.035). Studies from tertiary/teaching hospitals reported higher prevalence (6.1%) than community hospitals (2.8%), but the between group difference was not statistically significant (*P* = 0.086). Overall, these findings indicate that diagnostic intensity and study rigor significantly impact stroke detection rates and may partially explain the high heterogeneity observed between studies.

**Table 2:** Subgroup analysis of stroke prevalence in all emergency department dizziness patients.

Subgroups	No. of studies	No. of participants	Prevalence, % (95% CI)	I^2^	Heterogeneity between groups
Overall	21	158583	5.5 (4.1, 7.1)	99.14	NA
Study year					0.003
≤ 2015	7	24350	3.1 (1.8, 4.6)	90.97	
> 2015	14	134233	6.9 (4.9, 9.2)	99.32	
Continent					0.016
North America	8	122036	3.0 (1.4, 5.1)	99.54	
Asia	9	32383	6.8 (4.0, 10.4)	98.24	
Europe	4	4164	10.5 (4.3, 19.0)	97.93	
Study design					0.035
Prospective	6	3168	9.4 (0.49, 15.1)	93.36	
Retrospective	15	155415	4.5 (3.1, 6.1)	99.36	
Source of the patient					0.086
Tertiary/Teaching Hospitals	17	58911	6.1 (4.4, 8.1)	98.45	
Community Hospitals	3	21679	2.8 (0.6, 6.3)	NA	
Diagnostic method					<0.001
MRI with or without follow–up	2	210	24 (18.4, 30.0)	NA	
Clinical follow–up/Composite assessment	15	49940	5.5(3.7, 7.6)	98.52	
Coding/Discharge summary only	4	108433	2.4 (0.9, 4.5)	99.53	
Imaging Rate					<0.001
< 100% or Unspecified	19	158373	4.6 (3.3, 6.1)	99.19	
100%	2	210	24.0 (18.4, 30.0)	NA	
Study sample size					0.002
≥ 500	14	156787	3.0 (1.9, 4.3)	99.30	
< 500	7	1796	14.9 (6.2, 26.3)	97.14	

CI: confidence interval; NA: not available.

### Stroke prevalence in ED isolated dizziness patients

**Fig 3:**
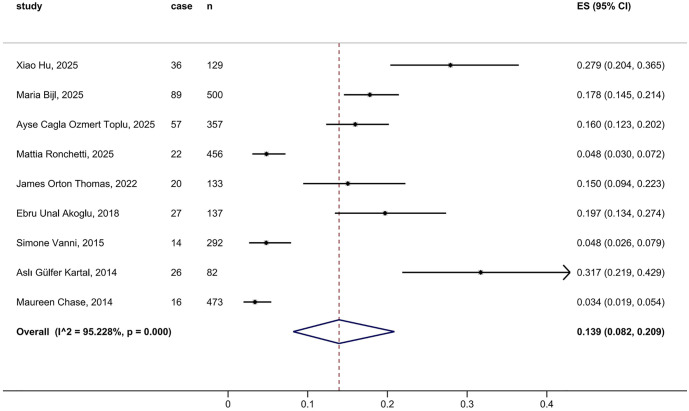
Pooled prevalence of stroke among ED patients with isolated dizziness.

Pooled analysis of 9 studies involving 2,559 individuals showed that the overall pooled stroke prevalence among ED patients with isolated dizziness was 13.9% (95% CI, 8.2–20.9; I² = 95.23) (Fig 3). The funnel plot appeared asymmetric (S3A Fig), but Egger’s test did not indicate significant publication bias among the 9 included studies (*P* = 0.369). Sensitivity analysis using the leave-one-out method did not reveal any study the exclusion of which caused a directional change in the results (S3B Fig).

All 9 studies reported the number of ischemic and hemorrhagic strokes separately. The pooled prevalence of ischemic stroke in ED isolated dizziness patients was 13.2% (95% CI, 7.5–20.2; I² = 95.46) (S4A Fig), whereas hemorrhagic stroke prevalence was 0.5% (95% CI, 0.2–0.9; I² = 0.0) (S4B Fig). Additionally, 5 studies reported the number of TIA cases, yielding a pooled prevalence in ED isolated dizziness patients was 2.6% (95% CI, 0.1–7.9; I² = 94.93) (S4C Fig).

### Subgroup analysis of stroke in isolated dizziness patients

Subgroup analysis of 9 studies (n = 2,559) showed highly significant differences between studies from different location (*P* < 0.001). For ED isolated dizziness patients, Asia showed the highest stroke prevalence (22.9%), followed by Oceania (15.0%), Europe (8.4%), and North America (3.4%). There were also extremely significant differences based on the level of the medical center (*P* < 0.001). Stroke centers (15.7%) and tertiary/teaching hospitals (15.5%) had similar and relatively high prevalence rates, while community hospitals had a significantly lower rate (4.8%). In terms of study design, retrospective designs reported a significantly higher prevalence (19.7%) than prospective studies (11.8%), with a between-group difference *P* = 0.045. Regarding diagnostic methods and imaging coverage, although not statistically significant, studies using MRI (with or without follow-up) had a stroke prevalence (19.5%) almost double that of studies relying on clinical follow up/composite assessment (10.0%). Similarly, studies with 100% imaging coverage had a higher prevalence (19.8%) than those with inadequate or unspecified coverage (11.3%), but the between group difference was not significant (*P* = 0.136). Furthermore, there were no significant differences in prevalence based on sample size or publication year (Table 3).

**Table 3:** Subgroup analysis of stroke prevalence in emergency department patients with isolated dizziness.

Subgroups	No. of studies	No. of participants	Prevalence, % (95% CI)	I^2^	Heterogeneity between groups
Overall	9	2559	13.9 (8.2, 20.9)	95.23	NA
Study year					0.425
≤ 2015	3	847	10.2 (1.9, 23.8)	NA	
> 2015	6	1712	15.9 (9.6, 23.5)	93.14	
Continent					<0.001
Europe	3	1248	8.4 (2.1, 18.2)	NA	
Asia	4	705	22.9 (15.9, 30.7)	79.24	
Oceania	1	133	15.0 (9.4, 22.3)	NA	
North America	1	473	3.4 (1.9, 5.4)	NA	
Study design					0.045
Prospective	7	1930	11.8 (6.1, 18.9)	94.5	
Retrospective	2	629	19.7 (16.6, 22.9)	NA	
Source of the patient					<0.001
Tertiary/Teaching Hospitals	6	1613	15.5 (7.2, 26.3)	96.25	
Stroke Center	2	490	15.7 (12.6, 19.1)	NA	
Community Hospitals	1	456	4.8 (3.0, 7.2)	NA	
Diagnostic method					0.062
MRI with or without follow–up	4	709	19.5 (13.9, 25.8)	71.81	
Clinical follow–up/Composite assessment	5	1850	10.0 (3.8, 18.6)	96.46	
Imaging Rate					0.136
< 100% or Unspecified	6	1987	11.3 (5.1, 19.6)	96.07	
100%	3	572	19.8 (12.1, 28.8)	NA	
Study sample size					0.181
≥ 300	4	1786	9.5 (3.4, 18.2)	96.63	
< 300	5	773	18.4 (8.5, 31.0)	93.79	

CI: confidence interval; NA: not available.

### Diagnostic performance of bedside tools

Four studies reported the diagnostic performance of bedside tools for diagnosing stroke in ED dizziness patients, including TriAGe + , ABCD2, HINTS, and STANDING. Table 4 summarizes their performance metrics along with brief descriptions of each tool’s components and clinical application. Due to the limited number of studies and methodological heterogeneity, we provide a qualitative synthesis of the available evidence. Measured by the Area under the curve (AUC) for overall discriminative ability, TriAGe+ and HINTS performed excellently, both with AUCs greater than 0.88, while the ABCD2 score showed relatively poor discriminative ability (AUC = 0.71). The highest sensitivity was observed for the HINTS examination [100% (78.2–100.0)] and the TriAGe+ score at the low cutoff point (>5) [96.4% (87.3–99.5)], indicating an extremely low risk of missing a stroke with these methods. In contrast, both the TriAGe+ at the high cutoff point (>10) and ABCD2 score had sensitivities below 70%. Regarding specificity, the TriAGe+ high cutoff point (>10) performed exceptionally well [99.8% (98.6–100.0)], indicating an extremely low false-positive rate. Likelihood ratio analysis provides more direct clinical utility. The TriAGe+ high cutoff point (>10) produced a very high positive likelihood ratio (+LR) (237.3), suggesting that a patient scoring above 10 has a substantially increased probability of having a stroke. HINTS, STANDING, and the TriAGe + low cutoff point (>5) all had very low negative likelihood ratios (-LR) (0.0–0.1). According to evidence-based medicine principles, a negative result with these tools can significantly reduce the post-test probability, allowing for high confidence rule out of stroke (Table 5).

**Table 4:** Performance metrics and clinical application of bedside diagnostic tools for ED patients with dizziness.

Tool	Description and Components	Interpretation	Target Population
TriAGE+	8-variable score: triggers, atrial fibrillation, male sex, hypertension, brainstem/cerebellar dysfunction, focal weakness/speech impairment, dizziness (non-vertigo), no history of vertigo	Higher score indicates higher stroke risk	All dizzy patients
HINTS	3-step oculomotor examination: head impulse test, nystagmus type, test of skew	Any central sign (normal head impulse, direction-changing nystagmus, skew deviation) → central etiology	Only applicable for patients with nystagmus (acute vestibular syndrome)
STANDING	4-step algorithm: ① spontaneous/positional nystagmus differentiation ② nystagmus direction ③ head impulse test ④ standing balance test	Sequential interpretation; any abnormality suggests central etiology	Patients with acute vertigo/dizziness
ABCD2	5-variable score: age, blood pressure, clinical features, duration, diabetes	Score ≥4 suggests increased stroke risk (but limited performance in dizzy patients)	Originally developed for TIA risk stratification

**Table 5:** Performance metrics of four bedside diagnostic tools for stroke in emergency department dizziness patients.

Study	Diagnostic tools	Cut-off point	AUC	Sensitivity	Specificity	LR+	LR-
Adrian Ho–Kun Yu, 2024	TriAGe+	>5	0.90 (0.87–0.93)	96.4 (87.3–99.5)	42.4 (37.5–47.4)	1.7 (1.5–1.8)	0.09 (0.02–0.3)
		>10		59.3 (45.0–72.4)	99.8 (98.6–100.0)	237.3 (33.1–1704.0)	0.41 (0.3–0.6)
Ayse Cagla Ozmert Toplu, 2025	TriAGe+	≥ 10	0.88 (0.84–0.91)	46.6 (33.3–60.1)	96.3 (93.5–98.1)	12.7 (6.7–24.1)	0.55 (0.4–0.7)
	ABCD2	≥ 4	0.71 (0.63–0.73)	65.5 (51.9–77.5)	68.6 (63.0–73.8)	2.08 (1.6–2.7)	0.50 (0.4–0.7)
	HINTS	/	0.88 (0.80–0.94)	100 (78.2–100.0)	85.9 (75.6–93.0)	7.10 (4.0–12.6)	0.0 (–)
Mattia Ronchetti, 2025	STANDING	/	/	88.20%	91.60%	10.4 (6.6–16.6)	0.1 (0.0–0.5)
Simone Vanni, 2015	STANDING	/	/	92.9% (13/14)	96.4 (81/84)	/	/

AUC: area under the curve; LR + : positive likelihood ratio; LR-: negative likelihood ratio.

In summary, HINTS and STANDING demonstrated the best-balanced performance for both rule-out (high sensitivity, low -LR) and rule-in (high specificity, high +LR) stroke. TriAGe+ score can be adapted for different clinical purposes based on the cutoff value: the low cutoff (>5) is suitable for high-sensitivity screening, while the high cutoff (>10) is suitable for high-specificity confirmation. ABCD2 score has limited diagnostic value in this context.

## Discussion

This systematic review and meta-analysis comprehensively assessed stroke risk among ED patients presenting with dizziness, particularly those with isolated dizziness. The pooled prevalence of stroke was 5.5% among all ED dizziness patients, while the prevalence in isolated dizziness was markedly higher at 13.9%. Although isolated dizziness is often presumed to be benign, our findings indicate a substantially elevated stroke risk in this subgroup. Further evaluation of bedside diagnostic tools for stroke in dizzy patients indicated that HINTS and STANDING possess strong diagnostic performance and can aid early stroke identification n the ED setting.

The higher stroke prevalence in isolated dizziness patients may be attributed to several factors. First, diagnostic intensity and imaging coverage are pivotal. Subgroup analysis clearly showed that, for both overall dizziness and isolated dizziness patients, studies using MRI as the diagnostic method had substantially higher stroke prevalence rates (all dizziness: 24.0%; isolated dizziness: 17.6%) than those relying on clinical follow-up or coding data, indicating the critical impact of diagnostic intensity on detection rates. Posterior-circulation (vertebrobasilar) strokes often present with isolated dizziness, vertigo, or imbalance as the initial or sole symptom, without typical focal neurological deficits [50]. Such patients are easily misclassified as having benign vestibular disorders, yet the underlying stroke risk remains substantial [51]. Furthermore, atypical presentation of posterior circulation strokes often leads to delays in diagnosis and treatment, resulting in poorer patient outcomes [52]. In our study, most isolated dizziness patients underwent MRI to rule out central causes, thereby enhancing the detection of minor or posterior circulation strokes. Second, patient source and regional differences contributed significant heterogeneity. Subgroup analysis found that the stroke risk in isolated dizziness patients was much higher in stroke centers (15.7%) and tertiary/teaching hospitals (15.5%) than in community hospitals (4.8%). Differences in case complexity, clinical vigilance, and diagnostic capabilities among different levels of healthcare institutions likely account for these variations in stroke confirmation rates [21,30]. Simultaneously, striking regional differences (Asia: 22.9% vs North America: 3.4%) may stem from various complex factors, including the overall burden of stroke, allocation of healthcare resources, prevalence of population risk factors, and differences in clinical practice patterns [53]. Additionally, studies have found that higher education levels are associated with lower stroke risk; higher education may influence stroke occurrence through health literacy and risk factor management, potentially partly explaining the differences in stroke prevalence between low middle income and high income countries [54,55]. The study design itself also influenced prevalence estimates. Retrospective studies reported higher prevalence than prospective studies, possibly because retrospective studies are more likely to include severe cases ultimately diagnosed with stroke, whereas prospective studies more comprehensively cover all consecutive patients presenting with dizziness.

Regarding diagnostic tools, HINTS and STANDING demonstrated excellent diagnostic performance in ED dizziness patients, particularly in the isolated dizziness population. The HINTS examination achieved a sensitivity of 100%, and its ability to rule out stroke (extremely low negative likelihood ratio) makes it an ideal screening tool. Multiple studies have confirmed that in patients with acute vestibular syndrome (AVS), HINTS outperforms the ABCD2 score for identifying stroke, even showing higher sensitivity than early MRI diffusion-weighted imaging in some cases [56,57]. The GRACE3 guidelines also recommend the use of HINTS for clinicians trained in its use in patients with nystagmus [58]. This emphasizes the importance of integrating standardized bedside examinations into ED triage process, especially when neuroimaging resources are limited or rapid decision making is required [59,60]. It should be noted that our synthesis of these tools is qualitative rather than a formal diagnostic meta-analysis, owing to the limited number of studies and methodological heterogeneity. Nevertheless, the clinical utility of these bedside tests remains relevant, and our summary provides a practical overview of their performance and applications.

This study has several limitations. First, there was substantial heterogeneity among the included studies, with I² values exceeding 95% in most analyses, indicating that between-study variability far exceeded that expected by chance. Although subgroup analyses identified several important sources of variation (e.g., diagnostic method, region, and patient source), considerable residual heterogeneity remained. Therefore, our findings are more informative for identifying patterns and sources of variation than for providing precise prevalence estimates applicable to specific clinical settings. Second, several studies, particularly the large sample retrospective studies in the all-dizziness group, relied on clinical coding or discharge summaries rather than standardized imaging confirmation, which may lead to an underestimation of the stroke prevalence. Third, the definition of isolated dizziness might have subtle differences across studies, potentially introducing classification bias. Fourth, the diagnostic criteria for TIA were not uniform across the included studies. This heterogeneity in outcome definition likely contributed to the substantial statistical heterogeneity observed in our TIA analysis and may affect the comparability of prevalence estimates across studies. Fifth, the HINTS examination, despite its high sensitivity and specificity, is only applicable to patients presenting with nystagmus and therefore does not cover the full spectrum of ED patients with dizziness, which limits the generalizability of its clinical applicability.

In conclusion, ED patients presenting with isolated dizziness have a relatively high risk of stroke, with risk estimates varying substantially across regions and levels of healthcare institutions. However, given the considerable heterogeneity and publication bias across the included studies, these estimates should be interpreted with appropriate caution. Bedside examination tools such as HINTS and STANDING demonstrate high diagnostic accuracy in appropriate patient subsets and may assist with early risk stratification. Future research should focus on prospective, multicenter designs, standardize the definition of isolated dizziness and the diagnostic gold standard, and further explore the optimal diagnostic pathway integrating bedside examinations with rapid neuroimaging protocols to minimize missed diagnoses and improve patient outcomes.

## Supporting information

S1 Fig Publication bias and sensitivity analysis for the prevalence of stroke. In all emergency department dizziness patients.
(DOCX)

S2 Fig Forest plots for the prevalence of stroke subtypes and TIA among. All emergency department patients with dizziness.
(DOCX)

S3 Fig Publication bias and sensitivity analysis for the prevalence of stroke. In emergency department patients with isolated dizziness.
(DOCX)

S4 Fig Forest plots for the prevalence of stroke subtypes and TIA among. Emergency department patients with isolated dizziness.
(DOCX)

S1 Table Complete search strategy. (PDF)

S2 Table Appraisal of papers using Joanna Briggs Institute (JBI) checklist. (PDF)
