# Supplementary material for: Dizziness in the emergency department and risk of stroke: A systematic review and meta-analysis
Source: PLoS One. 2026 Apr 8;21(4):e0346556. doi: 10.1371/journal.pone.0346556 (PMC13061258; doi:10.1371/journal.pone.0346556)
Supplement: S1 Fig — In all emergency department dizziness patients. (DOCX) [file pone.0346556.s001.docx]

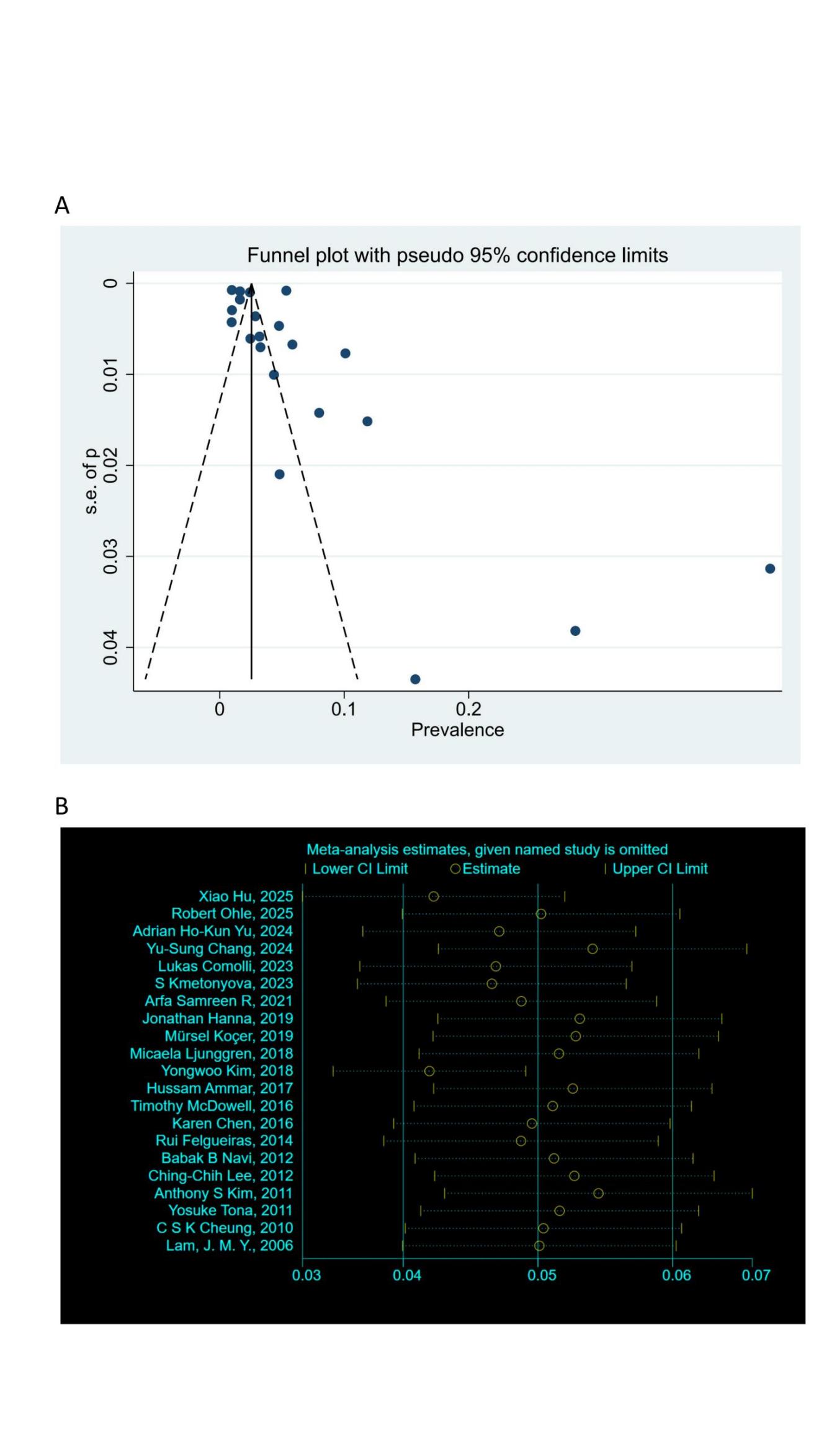


**Supplementary figure 1: Publication bias and sensitivity analysis for the prevalence of stroke in all emergency department dizziness patients.** A: Funnel plot for assessing publication bias in the prevalence of stroke among all emergency department dizziness patients; B: Sensitivity analysis (leave-one-out method) for the meta-analysis of stroke prevalence in all emergency department dizziness patients.
