# Supplementary material for: Dizziness in the emergency department and risk of stroke: A systematic review and meta-analysis
Source: PLoS One. 2026 Apr 8;21(4):e0346556. doi: 10.1371/journal.pone.0346556 (PMC13061258; doi:10.1371/journal.pone.0346556)
Supplement: S4 Fig — Emergency department patients with isolated dizziness. (DOCX) [file pone.0346556.s004.docx]

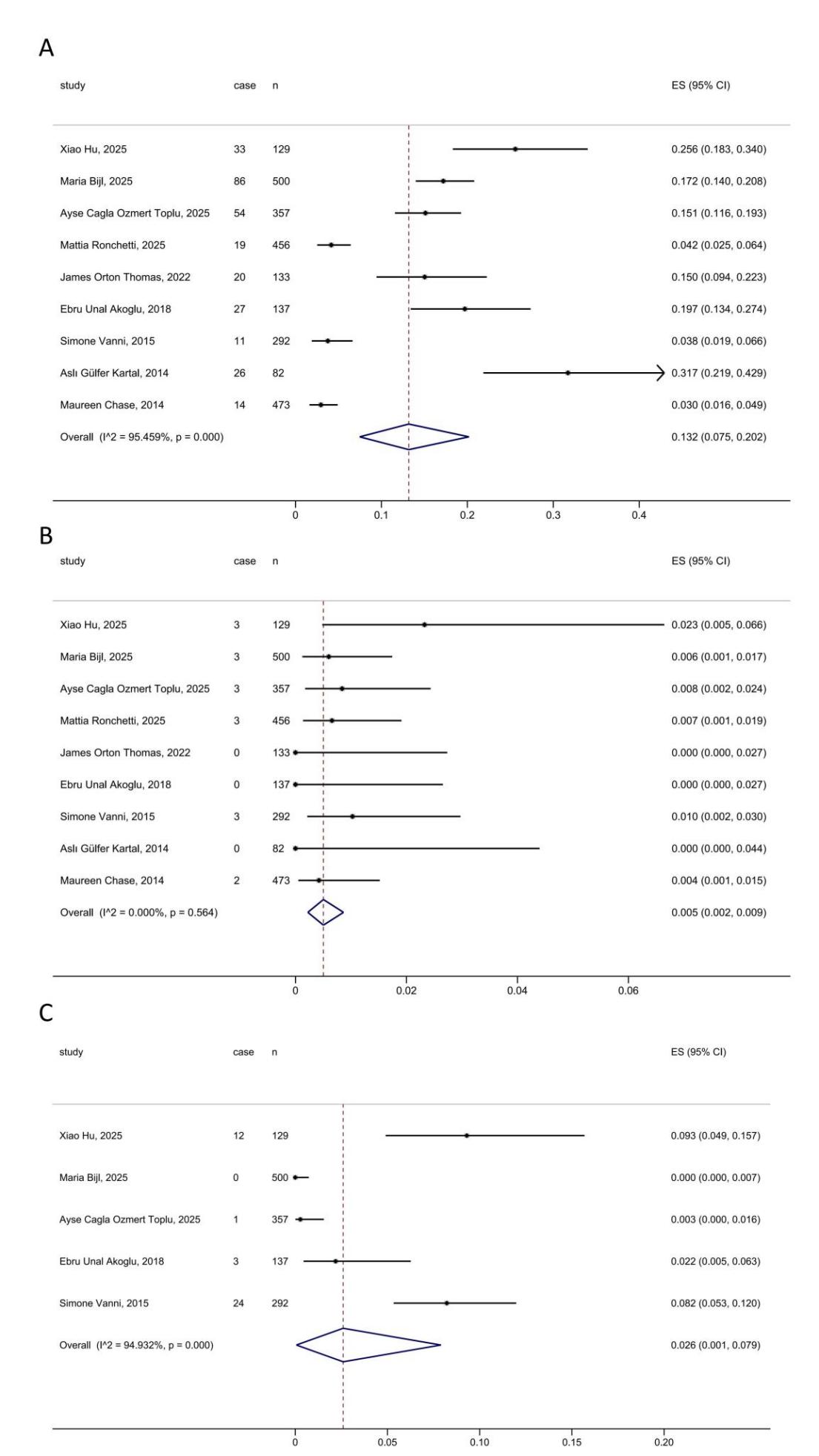


**Supplementary figure 4: Forest plots for the prevalence of stroke subtypes and TIA among emergency department patients with isolated dizziness.** A: Forest plot for the prevalence of ischemic stroke; B: Forest plot for the prevalence of hemorrhagic stroke; C: Forest plot for the prevalence of transient ischemic attack.
