# Supplementary material for: Dizziness in the emergency department and risk of stroke: A systematic review and meta-analysis
Source: PLoS One. 2026 Apr 8;21(4):e0346556. doi: 10.1371/journal.pone.0346556 (PMC13061258; doi:10.1371/journal.pone.0346556)
Supplement: S1 Table — (PDF) [file pone.0346556.s005.pdf]

Supplementary Table 1: Complete search strategy

1. pubmed

Search number	Query	Results
#1	(("Emergency Service, Hospital"[Mesh] OR "Emergency Department" OR "ED") AND ("Dizziness"[Mesh] OR "Vertigo"[Mesh] OR "dizzy" OR "vertigo")) AND ("Stroke"[Mesh] OR "Cerebrovascular" OR "Brain Ischemia"[Mesh] OR "Intracranial Hemorrhages"[Mesh] OR "TIA" OR "Transient Ischemic Attack" OR "cerebral infarct" OR "ischemic stroke" OR "hemorrhagic stroke") AND (2000:2025[pdat])	245

Search deadline 2025.7.30

2. Cochrane

Search number	Query	Results
#1	MeSH descriptor:[Emergency Service,Hospital] explode all trees	4,053
#2	(Emergency Department):ti,ab,kw OR (ED):ti,ab,kw	24,026
#3	#1 or #2	24,817
#4	MeSH descriptor: [Dizziness] explode all trees	1,066
#5	(Vertigo):ti,ab,kw OR (dizzy):ti,ab,kw	20,758
#6	#4 or #5	20,759
#7	MeSH descriptor: [Stroke] explode all trees	18,235
#8	(Cerebrovascular):ti,ab,kw OR (Brain Ischemia):ti,ab,kw OR (cerebral infarct):ti,ab,kw OR (TIA):ti,ab,kw OR (Transient Ischemic Attack):ti,ab,kw OR (ischemic stroke):ti,ab,kw OR (hemorrhagic stroke):ti,ab,kw	48,004
#9	#7 or #8	55,915
#10	#3 and #6 and #9	24

Search deadline 2025.7.31

3. Embase

Search number	Query	Results
#1	'emergency ward'/exp	260,491
#2	'Emergency Department':ab,ti OR 'ED':ab,ti	317,506
#3	#1 OR #2	419,496
#4	'dizziness'/exp	119,976
#5	'Vertigo':ab,ti OR 'dizzy':ab,ti	27,006
#6	#4 OR #5	141,458
#7	'Stroke'/exp	515,636
#8	'Cerebrovascular':ab,ti OR 'Brain Ischemia':ab,ti OR 'cerebral infarct':ab,ti OR 'TIA':ab,ti OR 'Transient Ischemic Attack':ab,ti OR 'ischemic stroke':ab,ti OR 'hemorrhagic stroke':ab,ti	270,771
#9	#7 OR #8	660,384
#10	#3 AND #6 AND #9	1,073

Search deadline 2025.7.31

4.Web of science

Search number	Query	Results
#1	(TS=(Emergency Department) OR AB=(Emergency Department OR ED OR emergency ward)) AND (TS=(dizziness) OR AB=(dizziness OR Vertigo OR dizzy)) AND (TS=(Stroke) OR AB=(Stroke OR Cerebrovascular OR Brain Ischemia OR cerebral infarct OR TIA OR Transient Ischemic OR ischemic stroke OR hemorrhagic stroke))	452

Search deadline 2025.7.31
