# Supplementary material for: Dizziness in the emergency department and risk of stroke: A systematic review and meta-analysis
Source: PLoS One. 2026 Apr 8;21(4):e0346556. doi: 10.1371/journal.pone.0346556 (PMC13061258; doi:10.1371/journal.pone.0346556)
Supplement: S2 Table — (PDF) [file pone.0346556.s006.pdf]

Supplementary Table 2: Appraisal of papers using Joanna Briggs Institute (JBI) checklist.

Study	Was the sample frame appropriate to address the target population?	Were study participants sampled in an appropriate way?	Was the sample size adequate?	Were the study subjects and the setting described in detail?	Was the data analysis conducted with sufficient coverage of the identified sample?	Was the condition measured in a standard, reliable way for all participants?	Were valid methods used for the identification of the condition?	Was there appropriate statistical analysis?	Was the response rate adequate, and if not, was the low response rate managed appropriately?	Total score
Robert Ohle, 2025	Y	Y	Y	Y	Y	Y	Y	Y	Y	9
Adrian Ho-Kun Yu, 2024	Y	N	Y	Y	Y	Y	Y	Y	Y	8
Yu-Sung Chang, 2024	Y	Y	Y	Y	Y	N	Y	Y	N/A	7
Lukas Comolli, 2023	Y	Y	Y	Y	Y	Y	Y	Y	Y	9
S Kmetonyova, 2023	Y	Y	N	Y	Y	Y	Y	Y	Y	8
Arfa Samreen R, 2021	Y	Y	N	Y	Y	Y	Y	Y	N	7
Jonathan Hanna, 2019	Y	Y	Y	Y	Y	N	Y	Y	N/A	7
Mürsel Koçer, 2019	Y	Y	Y	Y	Y	N	N	Y	Y	7
Micaela Ljunggren, 2018	Y	Y	Y	Y	Y	Y	N	Y	N/A	7
Yongwoo Kim, 2018	Y	Y	Y	Y	Y	N	N	Y	N/A	6
Hussam Ammar, 2017	Y	Y	Y	Y	Y	Y	Unclear	Y	N/A	7
Timothy McDowell, 2016	Y	Y	Y	Y	Y	Y	N	Y	N/A	7
Karen Chen, 2016	Y	Y	Y	Y	Y	Y	Unclear	Y	N/A	7
Rui Felgueiras, 2014	Y	Y	Y	Y	Y	Y	Y	Y	Y	9
Babak B Navi, 2012	Y	Y	Y	Y	Y	Y	Y	Y	Y	9
Ching-Chih Lee, 2012	Y	Y	Y	Y	Y	N	Unclear	Y	Y	7
Anthony S Kim, 2011	Y	Y	Y	Y	Y	Y	Y	Y	N/A	8
Yosuke Tona, 2011	Y	Y	Y	Y	Y	Y	N	Y	N/A	7
C S K Cheung, 2010	Y	Y	Y	Y	Y	Y	Y	Y	Y	9
Lam, J. M. Y., 2006	Y	Y	Y	Y	Y	Y	Y	Y	Unclear	8
Maria Bijl, 2025	Y	Y	Y	Y	Y	Y	Y	Y	N/A	8
Ayse Cagla Ozmert Toplu, 2025	Y	Y	Y	Y	Y	Y	Y	Y	Y	9
Mattia Ronchetti, 2025	Y	Y	Y	Y	Y	Y	Y	Y	Y	9
James Orton Thomas, 2022	Y	Y	N	Y	Y	Y	Y	Y	Y	8
Andreas Zwergal, 2020	Y	Y	Y	Y	Y	Y	Y	Y	N	8
Ebru Unal Akoglu, 2018	Y	Y	N	Y	Y	Y	Y	Y	N	7
Simone Vanni, 2015	Y	Y	Y	Y	Y	Y	Y	Y	Y	9
Aslı Gülfer Kartal, 2014	Y	Y	Unclear	Y	Y	Y	Y	Y	N	7
Maureen Chase, 2014	Y	Y	N	Y	Y	Y	Y	Y	Y	8

Y: yes, N: no, N/A: not applicable. Unclear: no relevant information provided.
